# Enzymatic Production of Sustainable Aviation Fuels from Waste Feedstock

**DOI:** 10.3390/molecules30234648

**Published:** 2025-12-03

**Authors:** Maria Mero, Vasiliki Mesazou, Elissavet Emmanouilidou, Nikolaos C. Kokkinos

**Affiliations:** 1 Department of Chemistry, School of Sciences, Democritus University of Thrace, Ag. Loukas, 654 04 Kavala, Greece; 2 Petroleum Institute, Democritus University of Thrace, Ag. Loukas, 654 04 Kavala, Greece; 3 Hephaestus Laboratory, School of Sciences, Democritus University of Thrace, Ag. Loukas, 654 04 Kavala, Greece

**Keywords:** sustainable aviation fuel, biojet, enzymes, fermentation, hydrolysis

## Abstract

The continuous fossil fuel exhaustion, as well as the increasing environmental challenges that are occurring globally, has underscored the need for research on alternative pathways of producing biofuels that will minimize aviation emissions over the next decades. The present review explores the employment of diverse waste sources as feedstock and enzymes as catalysts as environmentally friendly methods for producing sustainable aviation fuels (SAF). To achieve this goal, a comprehensive review was conducted using the Preferred Reporting Items for Systematic Reviews and Meta-Analyses. The results demonstrated that waste feedstocks catalyzed by enzymes represent an innovative alternative for SAF production. Specifically, the combination of enzymatic hydrolysis and microbial fermentation demonstrated considerable effectiveness in transforming complex waste feedstocks, such as lignocellulosic biomass, municipal solid waste, and food waste, into SAF precursors, including bio-isobutene and fatty acid methyl esters. Moreover, employing *Chlorella variabilis* fatty acid photodecarboxylase enzymes for photoenzymatic decarboxylation demonstrated significant conversion efficiency, particularly under gentle conditions, low energy consumption and remarkable selectivity. However, further research and development of the reviewed methods are necessary to enable the industrialization of these technologies.

## 1. Introduction

Greenhouse gas (GHG) emissions are primarily driven by human activities, including energy production, industrial processes, agriculture, and transportation. Transportation is responsible for a significant portion of global GHG emissions, primarily due to the burning of fossil fuels such as gasoline, diesel, and jet fuel. The sector contributes approximately 20–25% of global CO_2_ emissions, with road vehicles accounting for the largest share [1]. According to the International Energy Agency (IEA) [2], in 2023, aviation emissions increased to more than 90% of their 2019 pre-pandemic high level. As demand for air travel recovered in 2022 and 2023, emissions rose in all regions except Russia, reaching about 950 Mt CO_2_. Under the Net Zero Scenario, international aviation emissions could be reduced to 570.56 Mt CO_2_ by 2030. As a result, significant research on alternative and eco-friendly jet fuels is expected to continue in the near future. This would include using sustainable aviation fuels (SAF), which are notably produced from organic waste, including used cooking oil, agricultural residues, and food scraps. These resources can alleviate environmental degradation and provide an ecological alternative to fossil fuels [3]. Alongside other areas, several technologies for SAF production are being researched and developed, and among them, enzymatic treatments appear to be of special interest. Under the main scenario of IEA [4], demand for SAF is expected to rise to around 9 billion liters (0.3 EJ) by 2030, accounting for approximately 2.0% of global jet fuel demand. The ReFuelEU Aviation Act in the European Union mandates SAF blending requirements of 2% for 2025 and 6% by 2030. By 2030, a significant portion of Europe’s SAF demand is expected to be met by local production, with almost 4 billion liters of SAF capacity already in advanced development. The majority of additional demand for residual and vegetable oils will meet ReFuelEU feedstock standards, which include intermediate crops or marginal land. In North America, current SAF production and projects in advanced development phases are projected to yield a capacity of 3.3 billion liters by 2030. The projected SAF regulations in Japan (10%) and Singapore (1% by 2026) will generate an additional global demand of 1.4 billion liters (0.05 EJ). Recent studies have proven that oils, lignocellulosic biomass and other organic waste materials can be converted into biofuels through enzymatic hydrolysis, photoenzymatic decarboxylation, and fermentation [5,6,7]. Compared to highly energy-intensive thermochemical techniques, enzymatic processes can operate in mild conditions, such as lower temperatures and pressures [8]. Under these conditions, we can also utilize more challenging feedstocks that are typically difficult to decompose using conventional techniques [9]. However, despite the potential, before enzymatic SAF becomes a widely used substitute for large-scale industrial applications, challenges such as enzyme stability and the overall cost-effectiveness of the processes must be overcome [10]. The strict greenhouse gas (GHG) emission requirements set by the International Civil Aviation Organization (ICAO) have complete control over the bio-jet fuel market, and aviation companies that fail to comply with the rules face fines. Consequently, the use of bio-jet fuels has a significant impact on the corporate profit margin. The cost of the feedstock, its availability, the volatility of conventional jet fuel, and the viability of commercializing production systems all affect the price of bio-jet fuel [3].

The ASTM (American Society for Testing and Materials) has certified eight pathways to produce SAF. These pathways ensure that SAF meets stringent safety, performance, and compatibility standards with existing aviation engines and infrastructure. Hydroprocessed Esters and Fatty Acids-Synthetic Paraffinic Kerosene (HEFA-SPK) is one of the most mature and widely used pathways for producing Sustainable Aviation Fuel (SAF), involving the conversion of lipid-based feedstocks, such as waste cooking oils, animal fats, and vegetable oils, into jet fuel through hydroprocessing. Other promising technologies for converting waste biomass to SAF include the Alcohol-to-Jet (ATJ) and the Fischer-Tropsch (FT) pathways. ATJ involves several steps, starting with the enzymatic conversion of waste biomass into sugars, followed by fermentation to alcohols, and finally, upgrading the alcohols into jet fuel through catalytic processes. FT synthesis can utilize various biomass sources, including agricultural residues, wood, or waste materials, to convert syngas (a mixture of carbon monoxide and hydrogen) into liquid hydrocarbons, which can then be refined into biofuels, such as biojet fuel [1,3,11].

SAF, like Jet A-1, contains hydrocarbons. However, the quantity and kind may vary significantly. SAF ought to meet ASTM D1655 [12] and ASTM D7566 [13] criteria for commercial air transport, similar to JET A-1 or JET A quality, such as fuel composition, flash point, smoke point, density, and corrosion tendency [14]. Conventional kerosene derived from crude oil (Jet A−1) with a carbon atom range from 8 to 16, comprises diverse species categorized into four chemical families: (i) n-alkanes or n-paraffins, (ii) iso-alkanes or iso-paraffins, (iii) cyclo-alkanes (naphthenes or cyclo-paraffins), and (iv) aromatics. Additionally, antioxidants, antistatic agents, and metal deactivators are examples of some fuel additives that may be present [15]. Aside from compositional variations, SAF and conventional aviation fuels exhibit distinct physical and chemical properties. For instance, the final boiling points of Jet A and Jet A-1 fuels, and SIP fuel, are limited to 300 °C, 255 °C, and 255 °C, respectively. Whereas, the ranges of the density at 15 °C for Jet A or Jet A−1 fuel and FT-SPK fuel are 775–840 kg/m^3^ and 730–770 kg/m^3^, respectively, all measured according to ASTM D7566 [13]. SAFs exhibit differences in atomization and combustion performance compared with conventional aviation fuels due to differences in volatility, viscosity, and thermal stability, which, to some extent, can influence CO, NO_x_, and unburnt hydrocarbons (UHC) emissions, even though engine operating conditions have a greater influence [16].

Enzymatic hydrolysis is a cutting-edge technology that utilizes waste feedstocks for producing biofuels selectively through enzymes that break down lipids into free fatty acids (FFAs) which are then converted into biojet fuels without generating unwanted byproducts [6]. In addition, it is an eco-friendly alternative to fossil-derived jet fuels, as it enables the recovery of high-value lipids without harsh chemicals. Moreover, photoenzymatic decarboxylation is an innovative biochemical process that uses light energy to activate specific enzymes, removing carboxyl groups from fatty acids or other carboxylic compounds. As a result, it produces valuable hydrocarbons, including alkanes and alkenes, as biofuels or as feedstocks for chemical production. This can be achieved under mild conditions, usually at ambient temperature and pressure, in contrast to conventional chemical decarboxylation methods. Thus, it minimizes energy inputs, making it a highly energy-efficient and eco-friendly process [17].

A key strategy for bio-based sustainable aviation fuel production from lipid derived from organic waste is the microbial lipid SAF pathway [18]. Those lipids, for example, can be derived after the recovery of anthocyanins [19] or by oleaginous microorganisms [20]. As lipid feedstocks, vegetable oils and biowaste are commonly used; nonetheless, they are inadequate to meet the growing demand for sustainable aviation fuels in the future. As a promising alternative to tackle this challenge, lignocellulosic waste that consists of non-edible plant materials such as agricultural residues, forestry byproducts, and dedicated energy crops, can notably enhance the sustainable production of bio-jet fuel while minimizing environmental footprint and competitiveness with food resources [21]. Eventually, municipal solid waste (MSW) which is conventionally managed through incineration or landfilling can be used as feedstock for producing biofuels while mitigating climate change [2]. This comprehensive review examines the potential to establish a circular bioeconomy within the aviation industry, with a main focus on converting waste materials through enzymatic processes into sustainable aviation fuels for the future. Given the ongoing interest in the utilization of waste biomass for biofuel production, the novelty of the review lies in combining waste biomass feedstocks with enzymatic processes to convert them into SAF.

## 2. Results and Discussion

The use of waste sources and the application of enzymes as catalysts are crucial for meeting the future demand for SAF, reducing costs, and minimizing the negative environmental impact [3]. Recent literature review and experimental and modeling data on SAF production from various waste materials using several technologies and different enzymes are comprehensively discussed in Section 2.1 and Section 2.2, respectively. The results demonstrate the effectiveness of enzyme and microbial pathways in converting waste into SAF. Enzymatic hydrolysis, combined with microbial fermentation, degrades complex substrates such as lignocellulosic biomass, food waste, and solid municipal waste into SAF precursors, including bio-isobutene and fatty acid methyl esters.

### 2.1. Review Studies for SAF Production via Enzymatic Processes of Waste Biomass

**Figure 1:**
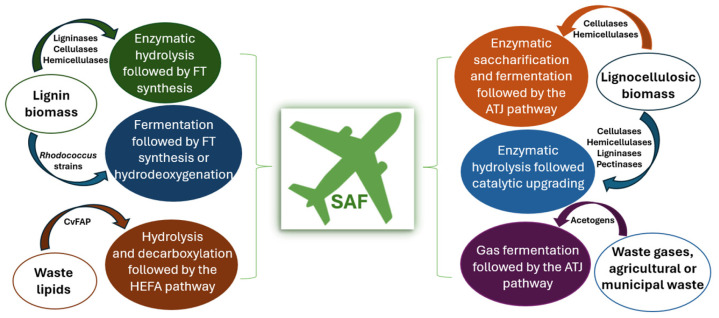
SAF production pathways from waste biomass via enzymatic processes.

Figure 1 illustrates SAF production pathways via enzymatic processes of waste biomass. Lignin, the most abundant natural source of aromatic compounds on a large scale, has a high energy density, making it a valuable renewable alternative to fossil fuels for directly producing various green fuels. Converting lignocellulosic biomass into aviation fuel precursors is crucial in producing fuel from biomass. Enzymatic hydrolysis is often combined with other pretreatment methods, playing a crucial role in lignocellulosic pretreatment by catalyzing the breakdown of lignin. However, enzymes can be costly and have a limited lifespan, as they often become denatured during pretreatment. Thermochemical techniques are advantageous over direct combustion as they emit less hazardous gases and produce valuable products during conversion. Fuels of high value and biojet can be refined via FT synthesis of combustible synthesis gases resulting from gasification. However, lignin gasification of lignocellulosic biomass is being evaluated at the pilot scale, with consequential investment costs and uncertainties in production expenses [22].

A promising method for producing fuels is bioconversion, which is environmentally benign and operates under mild conditions. *Rhodococcus* has attracted interest as a valuable microorganism with multiple catabolic capabilities for producing high-value products from biomass. Diverse methods for aviation fuel production have evolved, such as gasification-FT synthesis, thermal/catalytic cracking and hydrodeoxygenation, which is the most broadly implemented method for enhancing bio-aviation fuel quality. *Rhodococcus* strains can be utilized to convert lignin into lipids through fermentation, offering a hydrodeoxygenation strategy that does not allow lipid and residual lignin separation and thus has an important potential for lowering aviation fuel production cost and enhancing processes [23].

Guo et al. [24] proposed that the fatty acid photodecarboxylase (CvFAP) from *Chlorella variabilis* NC64A, which has been lately discovered, presents a viable route for producing carbon-neutral biofuels and fine chemicals. Triglycerides are hydrolyzed by lipase into long-chain fatty acids (LCFAs) and glycerol under mild conditions, with conversion rates of 85–100%. CvFAP then converts the resulting LCFAs into aliphatic hydrocarbons under visible light and normal pressure. This pathway streamlines biofuel production by serving as an alternative to the initial steps of HEFA.

The conversion of lignocellulosic biomass through enzymatic hydrolysis and fermentation presents a valuable solution for generating biofuels, especially bioethanol and biojet fuels. As described by Ashokkumar et al. [25], a suite of enzymes, including cellulases, hemisellulases and pectinases, through hydrolysis, break down the biomass into simpler sugar molecules. The resulting sugars are later fermented to produce bioethanol, which can be further refined into biojet fuel. Moreover, in some combined processes, enzymatic biomass breakdown is combined with syngas conversion via FT synthesis, leading to an increase in the overall yield of jet fuels. As part of the ATJ process, gas fermentation provides an alternative route to alcohol production that does not depend on sugars or biomass. Their ability to metabolize CO, CO_2_ and H_2_ into biomass and SAF precursors positions acetogenic microorganisms as valuable candidates for industrial gas fermentation applications. At present, LanzaTech uses CO-rich emissions from industrial and solid waste sources to produce ethanol. Rodriguez et al. [26] noted that the expanding H_2_ sector along with the waste gases generated by the national energy sector, could provide feedstocks for gas fermentation, elevating Australia’s role as a frontrunner in SAF production. Gas fermentation differs from biomass-based methods by converting waste CO and CO_2_, enabling a carbon-neutral or potentially carbon-negative process. This work utilized the metabolic adaptability of *Clostridium autoethanogenum* under anaerobic conditions to generate SAF intermediates, operating fermentations at 30 °C and 500 rpm. The authors focused on mitigating challenges, such as pathway compartmentalization and cofactor shortages, to improve the efficiency of isobutanol production.

Finally, Lynd et al. [27] examined enzymatic saccharification of lignocellulosic biomass, breaking down cellulose into sugars, followed by fermentation to produce ethanol, which is then converted into jet fuel through the ATJ pathway.

### 2.2. Experimental Studies for SAF Production via Enzymatic Processes of Waste Biomass

Table 1 presents recent experimental data on the enzymatic conversion of waste biomass into SAF.

**Table 1:** Experimental and modeling data for SAF production using various technologies and enzymes from various waste materials.

Waste Feedstock	Enzyme	Production Method/ SAF Pathway	Biojet Range Hydrocarbons or SAF Precursors	Highlights	Ref.
Recycled Paper	Cellulase	Enzymatic/Acid Hydrolysis followed by catalytic conversion and upgrading	Biojet: 76–83 million bbl/year	The minimum selling price estimate for paper-derived jet fuel is $3.97 per gallon. Direct cellulose hydrogenation could reduce capital and operating costs.	[28]
Food waste-derived CO_2_ and CH_4_	Photosynthetic and methanotrophic microorganisms	Anaerobic digestion/ Hydrocracking	Biojet: 0.137 kg CO_2_eq/MJ	By utilizing both CH_4_ and CO_2_ from biogas and taking advantage of potential subsidies for food waste disposal, bioroute-based SAF could become economically viable.	[29]
Food waste	*C*vFAP in the biphasic system	Photoenzymatic decarboxylation and anaerobic digestion	The decarboxylation products were 8-heptadecene (C17:1) and 6,9-heptadecadiene (C17:2)	CvFAP achieved the highest conversion of palmitic acid in a biphasic system using petroleum ether as the oil phase, reaching a rate 26.4 times higher than in a single-phase catalysis setup.	[30]
Palmitic acid as the model substrate for waste cooking oil	*Chlorella variabilis* fatty acid photodecarboxylase broken cells (CvFAP BCs) and CvFAP@*E. coli*	Photoenzymatic decarboxylation	Yields of 88.4% were obtained for pentadecane using CvFAP@*E. coli*, while CvFAP BCs achieved a yield of 95.4%.	The highest conversion rate of 17.2 mM·h^−1^ for CvFAP BCs was achieved, marking the highest rate ever reported.	[31]
Waste oils	CvFAP	Photoenzymatic decarboxylation	C15-C17 hydrocarbon biofuel	The CvFAP biocatalyst’s 100% selectivity for C15-C17 in biofuel production under mild conditions demonstrates the advantage of exclusive decarboxylation.	[32]
Garden waste	Mixed culture	Arrested anaerobic digestion and chain elongation	Caproic acid as a potential SAF precursor	High caproic acid yield was achieved under optimized conditions and gradual ethanol feeding, resulting in enhanced efficiency compared to batch fermentation.	[33]
Softwood residues	Modified bacterial *E. coli* strains	Enzymatic hydrolysis and fermentation/ATJ	Isobutene as SAF precursor	High GHG emission reduction (up to 80.1%) under optimal setups. The effective utilization of by-products, such as lignin, animal feed, and fertilizers, enhances system efficiency.	[34]
Pre- and post-consumer food waste	Engineered amylase	Simultaneous saccharification and fermentation	Bioethanol as SAF precursor: Yield: 61.2% to 87.6%	Thermal sterilization at an elevated liquefaction temperature significantly enhanced ethanol yields from pre-and post-consumer food waste, outperforming chemical decontamination methods.	[35]
Office paper waste	Yeast isolated from rotten banana	Enzymatic hydrolysis and fermentation/ATJ	Bioethanol as SAF precursor Total flux 253.06 g/m^2^·h	When integrated into a pervaporation system using an Amicon cell, the membrane effectively increased the bioethanol content from 30% to 63%.	[36]
Potato by-products	Alpha-amylase	ABE fermentation and catalytic upgrading/ATJ	Biojet: 10.7 total kt/y	Depending on the chosen feedstock and processing configuration, the GHG emission reduction potential of the innovative jet fuel was estimated to range between 41% and 52%.	[37]
Case 1: Wheat straw (WS) Case 2: Industrial cellulosic residue(ICR)	Cellulase	Enzymatic saccharification and fermentation/HDO of higher carbon alcohols	Biojet: 7.2 tonnes WS/ tonne biojet fuel (Case 1) Biojet: 16 tonnes ICR/ tonne biojet fuel (Case 2)	Monte Carlo risk analysis indicates a high likelihood of profitability, with a 96.66% probability for Case 1 and an even higher 99.99% probability for Case 2, given the current bio-jet fuel price of 15,000 CNY per tonne.	[38]
Palm Oil Mill Effluent	Immobilized lipase	Enzymatic Hydrolysis/ Hydrocracking	Biojet: 94% yield and 57.44% selectivity	High free fatty acid yield (90%), efficient hydrocarbon production using low catalyst loading	[39]
Sugarcane derived microbial oil	Oleaginous yeast	Aerobic fermentation/HEFA	Biojet: 2450 L/ha	SAF produced from microbial oil achieves a reduction of over 50% in GHG emissions compared to fossil fuels. Additionally, microbial oil derived from sugarcane yields four times higher SAF per unit area than soybean oil.	[40]
Paper sludge	Cellulase: Cellic CTec2 from Novozymes	Enzymatic hydrolysis, dehydration, and aldol condensation/ Hydroprocessing	More than 330 million gallons of SAF can be produced annually from the over 4 million dry tonnes of paper sludge available each year in the U.S.	The GWP of converting one dry ton of paper sludge to SAF is estimated to range from −584 to −636 kg CO_2_ eq per dry ton without ash utilization and from −873 to −925 kg CO_2_ eq per dry ton with ash utilization.	[41]
Lipid-rich wastewater	CvFAP	Photoenzymatic decarboxylation	Biojet: Production rate of 59.8 mM/h	Continuous photoenzymatic decarboxylation in a microfluidic photobioreactor yielded an impressive energy output of 33.6 kJ·g^−1^.	[42]
Corn Stover	Novozymes	Enzymatic hydrolysis and fermentation/ Catalytic upgrading	Biojet: 35 wt. % or 40.9% carbon-based yield	The jet fuel blendstock contains desirable n-alkanes, isoalkanes, and monocyclic cycloalkanes, along with undesirable alkynes and olefins.	[43]
Straw biomass	*C. beijerinckii* strain	Iron-catalyzed hydrogen peroxide pretreatment, enzymatic hydrolysis and fermentation/ ATJ	Bio-Jet: Conversion of 94.9% and selectivity of 77.3%	Fe-HP pretreatment enhanced sugar yield and concentration during enzymatic hydrolysis, facilitating more efficient fermentation and bio-jet fuel production.	[44]
Logging residues	Microbial strains	Enzymatic hydrolysis and fermentation/ ATJ	Biojet: 81,540 L/day and 102,600 L/day for Ethanol-to-Jet and Isobutanol-to-Jet pathways, respectively.	In the ATJ pathway, the minimum average selling price with co-product credit was lower for the Iso-BTJ pathway compared to the ETJ pathway.	[45]

Kubic et al. [28] have proposed a two-model approach to recycled paper as a feedstock for bio-jet fuel production, expanding on prior studies. A techno-economic analysis was conducted to evaluate the viability of enzymatic and acid hydrolysis for biofuel production. The study involved a six-step enzymatic hydrolysis process, a two-step acid hydrolysis process, and subsequent catalytic conversion and upgrading, including sugar hydrogenation to sugar alcohols and the transformation of sugar alcohols into hydrocarbons. The findings indicated that bio-jet fuel yields were 2.40 barrels per tonne for enzymatic hydrolysis and 2.36 barrels per tonne for acid hydrolysis. The direct hydrogenation of cellulose and hemicellulose into sugar alcohols is crucial for making the paper-to-jet fuel process economically viable. This approach can significantly reduce capital and operating costs. Nonetheless, existing limitations related to production costs and technical matters hinder the potential large-scale implementation of the process. Biogas, which consists of CO_2_ and CH_4_, can be produced from anaerobic digestion under mild conditions of food waste. The gases in biogas can serve as substrates for microorganisms, producing microbial lipids that can then be refined into SAF [46]. In their recent study, Zhang et al. [29] introduced a sustainable, carbon-neutral and cost-efficient method to produce SAF from food waste. The CO_2_ and CH_4_ from food-waste-derived biogas can be transformed by microalgae and methanotrophs into lipids that are later refined into SAF. Guo et al. [30] investigated food waste, where oil is separated from water, followed by photoenzymatic decarboxylation of waste oil (WO) lipase hydrolysate. WO was hydrolyzed by lipase to generate long-chain fatty acids (LCFA), which were then subjected to photoenzymatic decarboxylation. Meanwhile, non-oil components (NOC) were utilized as feedstock for anaerobic digestion (AD) to produce methane. CvFAP achieved a palmitic acid conversion rate 26.4 times higher than in single-phase catalysis in the biphasic system using petroleum ether as the oil phase. The WO hydrolysate catalyzed by CvFAP produced C17 hydrocarbons at a rate of 1.7 mM/h. Additionally, the same authors, for the first time, compared the catalytic performance of photoenzymatic decarboxylation reactions between recombinant *Escherichia coli* cells expressing CvFAP (CvFAP@*E. coli*) and broken cells (CvFAP BCs) [31]. The study found that CvFAP BC and CvFAP@*E. coli* exhibit similar behavior under appropriate conditions. Both reactions were conducted at 30 °C under blue light and in an anaerobic environment. Pentadecane was produced at 88.4% from CvFAP@*E. coli* and 95.4% from CvFAP BC. The highest conversion rate for CvFAP BC reported to date was 17.2 mM/h. Li et al. [32] developed a photoenzymatic method to decarboxylate FFAs from waste oils into hydrocarbon fuels, with a focus on producing biojet fuel by initially hydrolyzing waste oils to release FFAs. This step was followed by the enhancement of enzyme activity and an increase in alkane yield, through decarboxylation utilizing CvFAP under visible light. Outstanding hydrocarbon production with a rate of 18.4 mM·h^−1^ and an energy output of 204.3 kJ·L^−1^·h^−1^ was attained by this research, demonstrating high efficiency. Harahap et al. [33] investigated the conversion of caproic acid into (SAF. To achieve this, caproic acid was produced from green waste utilizing microbial chain elongation (CE) with arrested anaerobic digestion (AAD) at 37 °C, pH 7.2, and a 15% inoculum level, with stepwise ethanol addition to enhance its yield. This pathway demonstrated an innovative, high-efficiency, scalable approach to SAF precursor production from green waste, addressing feedstock volatility and the need for enhanced fermentation stability in industrial applications. Puschnigg et al. [34] introduced an advanced biorefinery model that converts softwood residues, such as sawdust, into SAF and valuable by-products. The process begins with enzymatic hydrolysis to extract sugars, which are then fermented using genetically engineered *E. coli* to produce bio-isobutene, a key SAF precursor. Subsequent oligomerization and hydrogenation steps yield SAF isoparaffins that comply with ASTM D7566 standards [13]. Environmental assessments indicate significant GHG emission reductions of up to 80.1% under the LI-B RES scenario (Lignin boiler, RES grid), which integrates renewable electricity and on-site lignin utilization for thermal energy.

Van Rooyen et al. [35] examined the use of pre- and post-consumer food waste to produce biojet fuel, utilizing optimized enzymes to advance process efficiency while integrating decontamination strategies. To eradicate microbial contaminants and enhance ethanol yields, food waste was pretreated with antimicrobial peptides (AMPs) or potassium metabisulfite (PMB). Moreover, fermentation was conducted using the concurrent saccharification and fermentation method, and the ATJ pathway was applied to convert to biojet fuel. Mansy et al. [36] explored the use of office paper waste to produce bioethanol through a sustainable integrated method of hydrolysis, fermentation and purification. Ethanol purity was increased by the implementation of a novel polymeric membrane composed of sulfonated polyvinyl chloride (SPVC) combined with poly(2-acrylamido-2-methyl−1-propanesulfonic acid) (PAMPS) to separate ethanol via pervaporation, resulting in increased concentration raising lab-prepared ethanol from 25% to 56% and bioethanol from 30% to 63%, with total flux values of 289.54 g·m−^2^·h^−1^ and 253.06 g·m−^2^·h^−1^, respectively. Moretti et al. [37] examined the potential of producing SAF via the ATJ pathway using potato by-products as feedstock through acetone-butanol-ethanol (ABE) fermentation, followed by catalytic upgrading. This approach resulted in 88% conversion to jet-range alkanes, with a minor fraction allocated for lubricant production. An analysis of economic and environmental impact was conducted for both the centralized and decentralized production models, which showed that the centralized approach resulted in 5% lower GHG emissions. In their study, Hong et al. [38] assessed two potential feedstocks, wheat straw (Case I) and industrial cellulosic residue (Case II), for the sustainable conversion of biomass to bioethanol, followed by the production of a hydrocarbon fuel from aqueous ethanol via C–C coupling and HDO. The results demonstrated that for Case I, the biomass feedstock consumption for bio-jet fuel production is 7.2 tonnes of wheat straw with a 5% moisture content, whereas for Case II, 16.0 tonnes of industrial cellulosic residue with a 45% moisture content per tonne of fuel, respectively.

Muanruksa et al. [39] investigated the utilization of palm oil mill effluent (POME) using a combination of enzymatic and thermochemical methods for the production of biojet fuel. The POME was first hydrolyzed under optimal conditions of 40 °C and 200 rpm using immobilized lipase, achieving a 90% FFA yield. Using hydrocracking, the resulting hydrolyzed product (HPOME) was treated with a palladium/alumina (Pd/Al_2_O_3_) catalyst at 400 °C and 10 bar hydrogen pressure for 1 h. Through the aforementioned process, FFAs were converted into hydrocarbons, producing mainly green kerosene with a 57.44% selectivity and 94% crude biofuel yield. This method outlined the effectiveness of industrial waste utilization and addressed ecological challenges associated with POME while producing valuable renewable fuels. Marchesan et al. [40] assessed the application of HEFA technology in SAF production from microbial oil (MO). By incorporating sugar and starch crops, as well as lignocellulosic waste, it expands the feedstock options for established SAF production. Due to the high productivity of sugarcane, averaging 76.3 tonnes per hectare, SAF production via MO-HEFA reaches 2450 L per hectare. This is nearly four times higher than the 630 L/ha yield of soybeans, which have an average productivity of 3.3 tonnes of soybean grains per hectare in Brazil, assuming a SAF yield of 1 L/kg oil. Utilizing more productive feedstocks helps minimize pressure on land conversion, thereby reducing both direct and indirect GHG emissions associated with land-use change. Lan et al. [41] conducted a cradle-to-grave life cycle assessment (LCA) of a SAF biorefinery in the U.S., utilizing a catalytic sugar upgrading pathway. The results indicate that selecting acetone instead of dioxane, wood pellets over natural gas, and biobased methyl ethyl ketone (MEK) over fossil-based MEK can significantly lower the life-cycle global warming potential (GWP) of SAF. However, while wood pellets help reduce GWP and fossil fuel depletion, their use increases environmental impacts in other categories due to emissions from wood combustion. Li et al. [42] achieved a maximum bio-aviation fuel production rate of 59.8 mM/h through the photodecarboxylation of FFAs extracted from lipid-rich wastewater into C1-shortened alkanes under blue light illumination. This presents a viable alternative for bio-aviation fuel production under mild conditions.

Affandy et al. [43] reported that high-titer 2,3-butanediol (2,3-BDO) fermentation at a 100 L scale, pretreated with nanofiltration, resulted in a reduction in impurity levels from 4.6 to 0.6 wt. %. A novel catalytic upgrading process was then developed to convert aqueous 2,3-BDO into a jet fuel blendstock candidate. The oligomerization of light olefins, demonstrated for over 270 h of time-on-stream (TOS), primarily produced dimers (C8–C10) and trimers (C13–C14). The resulting oligomerized product underwent hydrogenation and distillation to recover the jet fraction, yielding a mass fraction of 35% (corresponding to a carbon-based yield of 40.9%). Additionally, Zhu et al. [44] investigated the impact of iron ion-catalyzed hydrogen peroxide (Fe-HP) pretreatment on enzymatic hydrolysis, fermentation, and bio-jet fuel production from straw. The enzymatic hydrolysis and fermentation efficiency of straw biomass were notably improved after Fe-HP treatment, enabling the conversion of the feedstock into bio-jet fuels using a dual-functioning catalyst. This process resulted in a 94.9% high conversion rate and 77.3% jet fuel selectivity. Akter et al. [45] examined the use of forestry waste logging residues as feedstock for SAF production via the ATJ process, including both ETJ and Iso-BTJ pathways. Their study indicated that higher alcohol yield (0.33 L/kg) and lower carbon intensity (758 g CO_2_eq/L) are achieved through the ETJ pathway, whereas Iso-BTJ resulted in 0.24 L/kg and 976 g CO_2_eq/L, respectively. Although ETJ led to greater GHG savings, Iso-BTJ compensated by achieving higher daily SAF production.

From the results above, the highly promising and most widely used enzyme appears to be CvFAP, followed by cellulase. The first exhibits high performance, either in selectivity or conversion rate, when used with different types of waste feedstock in the photodecarboxylation process. On the other hand, cellulase was primarily applied to paper-related waste. This type of feedstock, along with food waste, appears to spark interest in SAF production research. As for the production pathways, enzymatic hydrolysis and photoenzymatic decarboxylation are the most commonly used to potentially produce SAF. In this review, it was observed that the combination of waste feedstock and an enzymatic process, delivering the highest yield of 95.4% and the highest conversion rate of 17.2 mM/h, was achieved by utilizing waste cooking oil together with CvFAP BCs under the photoenzymatic decarboxylation method. In addition, when the process mentioned above was repeated using CvFAP and waste oil, the study reported 100% selectivity for C15-C17 hydrocarbon biofuel. As for the environmental aspect, the pathway that achieved the highest GHG emission reduction, up to 80.1%, was anaerobic digestion and chain elongation of garden waste. The enzyme employed was a mixed culture that enabled conversion of the waste feedstock into caproic acid, a SAF precursor.

## 3. LCA and Economic Viability of SAF Produced via Enzymatic Processes

The LCA of SAF suggests that it has the potential to reduce GHG emissions by 26–93% compared to fossil-based jet fuel, disregarding land-use change effects. The steps that account for the majority of CO_2_ emissions in the SAF are feedstock cultivation and collection, as well as feedstock conversion and processing to jet fuels, with fuel transportation and feedstock transportation contributing the least to the core LCA value [47]. SAF produced from various feedstocks and techniques, including pyrolysis, FT, HEFA, and ATJ, can drastically reduce aviation industry GHG emissions by 41–89%. Because sulfur concentration and SO_2_ emissions are directly correlated, pure FT fuel can lower SO_2_ emissions from pure JP−8 by 92% because SAFs have a very low sulfur content [16]. A recent study conducted by Bhatt et al. [48] indicated that the life cycle GHG emissions from the woody FT pathway showed the greatest reduction at 86% compared to Jet A, assuming GHG emissions from conventional jet fuels are 85 g CO_2_ eq/MJ. In the ATJ pathway using crop residue as the feedstock, life-cycle GHG emissions were reduced by 67%. Lan et al. [41] found that converting 1 dry kg of waste paper sludge into SAF through ash removal, enzymatic hydrolysis, dehydration, aldol condensation, and hydroprocessing is more climate-friendly (−925 to −584 g CO_2_ eq/MJ) than landfilling without landfill gas recovery. Quiroz et al. [49] suggested using prospective LCA methods to evaluate the environmental impacts of advanced technologies still at the research and development stage for long-term estimation. Specifically, they highlighted that the miscanthus-FT pathway achieved a carbon intensity below 42 g CO_2_ eq/MJ under all the scenarios investigated. On the other hand, the corn-ATJ GWP distribution is centered between 55 and 60 g CO_2_ eq/MJ and extended to elevated emissions of 70 g CO_2_ eq/MJ. According to a recent review of LCA applications for biobased SAF production processes, there remains a scarcity of cradle-to-cradle LCAs that mimic a circular economy that includes SAF production. The capacity of a temporal LCA to address future uncertainties in consumer behavior, legislation, and regulations is also highly desired. Finally, as technological advancements in SAF conversion processes and feedstock production techniques continue, more LCAs are needed to evaluate the latest methods, enabling stakeholders to make informed decisions [50].

In terms of cost, the key factors are feedstock and capital costs across the SAF routes. Dedicated energy crops like switchgrass and miscanthus are more expensive (0.27–0.37 $/L) than biomass residues like forest and agricultural residues (0.18–0.28 $/L). As a result, residue feedstocks contribute less to the total harmonized SAF cost (15–17%) than energy crops (∼25%). In contrast, HEFA pathways have the lowest capital investment (541 MM$) and the highest returns (6.2–33.3 MJ SAF/kg biomass), resulting in the lowest capital cost per unit of SAF, accounting for 6–24% of the overall cost. In ATJ paths, the ethanol production stage incurs significant capital expenses, accounting for 39–42% of overall costs and becoming the primary contributor. In the case of pyrolysis, the comparatively low fuel production (2.47 MJ SAF/kg biomass) results in a high capital cost per unit of SAF, even though the capital input is very modest. ATJ routes demonstrate the least cost variance, since they operate within a tight yield range (0.11–0.13 kg feedstock/MJ). Among the ATJ choices, ATJ-MC and ATJ-SW have marginally higher production costs than ATJ-AR and ATJ-FR, reflecting their higher feedstock prices [51]. However, the increasing costs of renewable fuels have a minimal impact on consumers at current blending rates and under 2030 targets. If ReFuelEU Aviation targets are met, flying from Frankfurt to New York will cost an additional 2% due to a 5.3% sustainable aviation fuel blend rate and a 0.7% e-kerosene content [4].

## 4. Materials and Methods

**Figure 2:**
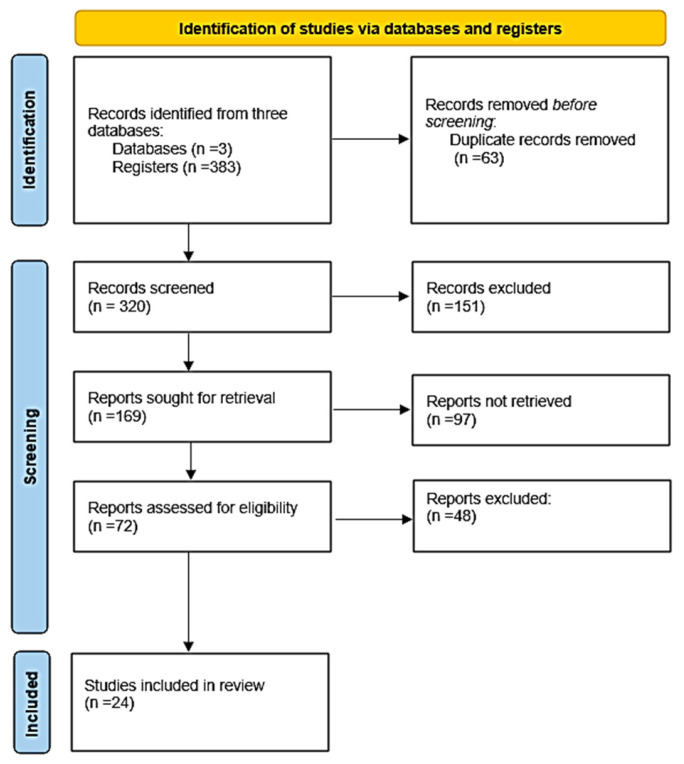
Flowchart of Preferred Reporting Items for Systematic Reviews and Meta-Analyses (PRISMA) of the comprehensive review study.

The method used to conduct the present comprehensive review is based on the Preferred Reporting Items for Systematic Reviews and Meta-Analyses (PRISMA) [52], as illustrated in Figure 2.

The Scopus, Google Scholar, and ScienceDirect databases were used to find relevant material. Database research was conducted from 14 November 2024 to 14 February 2025 to identify article titles, abstracts, and keywords published between 2014 and 2025. The search for relevant papers was conducted in the Scopus database using the term combination “sustainable AND aviation AND fuels”. In addition, the enhanced search was limited to articles and reviews published in English. Finally, an advanced search was performed using the following keywords: “waste OR enzyme OR production.” A total of 77 articles were identified. The Google Scholar database was also used for research using the following criteria: “waste” AND “enzyme” AND “production” AND “sustainable AND aviation AND fuel”, with an advanced search using the precise phrase “SAF production” between 2014 and 2025. A total of 213 papers were identified. Finally, in the Science Direct database, the keywords utilized were waste AND enzyme AND production, and an advanced search was again undertaken with the specific term “sustainable aviation fuels” to refine the search for article titles, abstracts, and keywords published between 2014 and 2025. A total of 93 articles were derived. In summary, 383 papers (77 from Scopus, 213 from Google Scholar, and 93 from Science Direct) were identified and exported from the databases to Zotero, containing the document title, abstract, authors, and year. Duplicates (63) were identified and eliminated. Furthermore, 151 records were excluded before screening because their titles did not align with the subject area. Specifically, either the title was unrelated to aviation biofuel production but linked to other biofuel production, feedstocks were not derived from waste materials, or enzymes were not used as catalysts when employing waste feedstocks. The following essential stage of record screening involved a cursory examination of each abstract and its associated keywords. Some abstracts did not meet the research criteria (97 records). Specifically, during the screening of the abstracts, the production route mentioned was unrelated to SAF and concerned only the production of biofuels used in other transportation sectors. Furthermore, although some abstracts mentioned waste feedstocks for SAF production, the conversion process was not enzymatic. Therefore, a total of 72 publications were deemed relevant to the current evaluation and thoroughly reviewed. Following a thorough investigation, 24 papers were included in this study, while 48 records were excluded because their results did not meet the research purpose regarding comparability with papers that referred to methods employing enzymes for SAF production from waste biomass. Specifically, upon thorough reading, some papers did not provide measurable data on yield or process performance. Waste feedstock was only a minor discussion point, not the subject of the study. Lastly, although some of the abstracts were accessible for screening, the full article texts were paywalled or otherwise inaccessible even after institutional access.

The quality and risk of bias of the studies included in the present review were assessed according to the PRISMA guidelines. Examining potential bias is crucial to the validity of the evidence supporting each process and to the transparency and reproducibility of the highlighted areas of each study. For this reason, each article was categorized as high, moderate, or low based on its methodological detail, experimental data and quantitative outputs. The scope of this section is to provide a clear and reliable foundation for the reported sources. Studies that included sufficient methodological reporting and clear experimental and quantitative results were classified as low risk, exhibiting reliability and minimizing uncertainty. In this category, 16 articles were classified, including the studies [26,28,29,30,31,32,33,36,38,39,40,42,43,44,45], which demonstrated well-organized experimental approaches and accounted for 69.6% of the analyzed articles. Studies that presented useful scientific information and contributed meaningfully, but did not thoroughly examine sensitivity parameters or experimental conditions, were categorized as moderate risk. Studies [34,35,37,41] are assigned to this category, representing 17.4% of the total. Studies that were more conceptual or had insufficient methodological detail were categorized as high risk. Finally, the studies [22,23,24,25], which primarily emphasize theoretical explanations, constitute 13% of the research examined.

## 5. Conclusions

Sustainable aviation fuels (SAF) are considered the most viable solution for the aviation industry to combat climate change, reduce emissions, and improve energy efficiency. Among the promising sources for bio-jet fuel production, waste feedstocks stand out, mainly catering waste, which has been identified as a significant risk factor. Methods that employ enzymes as catalysts to convert waste biomass feedstocks into biofuels offer an eco-friendly alternative to conventional chemical processes, whose conditions are often environmentally detrimental and intense. The approaches mentioned above provide high selectivity and energy efficiency, yielding specific hydrocarbon combinations while minimizing downstream process burdens when needed. The present review highlights the growing potential of enzymatic hydrolysis, photoenzymatic decarboxylation, and microbial lipid production as cutting-edge techniques for converting waste into sustainable aviation fuels (SAF) or their precursors. The combination of enzymatic hydrolysis and microbial fermentation has proven highly effective in converting lignocellulosic biomass, municipal solid waste, and food waste into SAF precursors, such as bio-isobutene, alcohols, and fatty acid methyl esters. Moreover, photoenzymatic decarboxylation demonstrates high conversion efficiency, especially under mild conditions, with low energy consumption and excellent selectivity. Nevertheless, further research on innovative developments in enzyme engineering, as well as the incorporation of biorefinery systems within circular economy frameworks, will be crucial to unlocking the full potential for large-scale SAF production and making a significant contribution to the decarbonization of the aviation industry.

## Data Availability

The data that support the findings of this study are available from the corresponding author upon reasonable request.

## Abbreviations

The following abbreviations are used in this manuscript:

AAD	Arrested anaerobic digestion
ABE	Acetone-butanol-ethanol
AD	Anaerobic digestion
AMP	Antimicrobial peptides
ATJ	Alcohol-to-Jet
ASTM	American Society for Testing and Materials
BC	Broken cells
2,3-BDO	2,3-butanediol
CE	Chain elongation
CVFAP	*Chlorella variabilis* fatty acid photodecarboxylase
ETJ	Ethanol-to-Jet
Fe-HP	Iron ion-catalyzed hydrogen peroxide
FFAs	Free fatty acids
FT	Fischer-Tropsch
GHG	Greenhouse gases
GWP	Global warming potential
HDO	Hydrodeoxygenation
HEFA-SPK	Hydroprocessed Esters and Fatty Acids-Synthetic
HPOME	Hydrolyzed palm oil mill effluent
ICAO	International Civil Aviation Organization
Iso-BTJ	Iso-Butanol-to-Jet
LCA	Life cycle assessment
LCFA	Long-chain fatty acids
LI-BRES	Lignin boiler, RES grid
MEK	Methyl ethyl ketone
MO	Microbial oil
MSW	Municipal solid waste
NOC	Non-oil components
PAMPS	Poly (2-acrylamido−2-methyl−1-propanesulfonic acid)
PMB	Potassium metabisulfite
POME	Palm oil mill effluent
PRISMA	Preferred Reporting Items for Systematic Reviews and Meta-Analyses
SAF	Sustainable aviation fuels
SPVC	Sulfonated polyvinyl chloride
TOS	Time-on-stream
UHC	Unburnt hydrocarbons
WO	Waste oil
